# Glutamate 338 is an electrostatic facilitator of C–Co bond breakage in a dynamic/electrostatic model of catalysis by ornithine aminomutase

**DOI:** 10.1111/febs.13215

**Published:** 2015-02-12

**Authors:** Binuraj R K Menon, Navya Menon, Karl Fisher, Stephen E J Rigby, David Leys, Nigel S Scrutton

**Affiliations:** Biotechnology and Biological Sciences Research Council/Engineering and Physical Sciences Research Council Centre for Synthetic Biology of Fine and Speciality Chemicals, Manchester Institute of Biotechnology, Faculty of Life Sciences, The University of ManchesterUK

**Keywords:** B_12_, dynamics, electrostatics, ornithine aminomutase, radical

## Abstract

How cobalamin-dependent enzymes promote C–Co homolysis to initiate radical catalysis has been debated extensively. For the pyridoxal 5′-phosphate and cobalamin-dependent enzymes lysine 5,6-aminomutase and ornithine 4,5-aminomutase (OAM), large-scale re-orientation of the cobalamin-binding domain linked to C–Co bond breakage has been proposed. In these models, substrate binding triggers dynamic sampling of the B_12_-binding Rossmann domain to achieve a catalytically competent ‘closed’ conformational state. In ‘closed’ conformations of OAM, Glu338 is thought to facilitate C–Co bond breakage by close association with the cobalamin adenosyl group. We investigated this using stopped-flow continuous-wave photolysis, viscosity dependence kinetic measurements, and electron paramagnetic resonance spectroscopy of a series of Glu338 variants. We found that substrate-induced C–Co bond homolysis is compromised in Glu388 variant forms of OAM, although photolysis of the C–Co bond is not affected by the identity of residue 338. Electrostatic interactions of Glu338 with the 5′-deoxyadenosyl group of B_12_ potentiate C–Co bond homolysis in ‘closed’ conformations only; these conformations are unlocked by substrate binding. Our studies extend earlier models that identified a requirement for large-scale motion of the cobalamin domain. Our findings indicate that large-scale motion is required to pre-organize the active site by enabling transient formation of ‘closed’ conformations of OAM. In ‘closed’ conformations, Glu338 interacts with the 5′-deoxyadenosyl group of cobalamin. This interaction is required to potentiate C–Co homolysis, and is a crucial component of the approximately 10^12^ rate enhancement achieved by cobalamin-dependent enzymes for C–Co bond homolysis.

## Introduction

In biology, reactions catalysed by B_12_-dependent enzymes are amongst the most unusual and complex in terms of activation energy requirements for homolysis of the organometallic C–Co bond [1–3]. Adenosylcobalamin (AdoCbl)-dependent enzymes homolyse C–Co bonds with a rate enhancement of approximately 10^12^ [4], and have evolved to control radical propagation effectively without enzyme inactivation or cellular damage [5–7]. Enzymatic contributions via ground-state destabilization (strain hypothesis and geometric distortion of AdoCbl) [8–10] or stabilization of the transition state (by electrostatic factors and van der Waals interactions) have been used to explain the C–Co bond activation and rate enhancement of homolysis achieved by enzymes [11,12]. The link between enzyme dynamics and catalytic rate enhancement in AdoCbl systems is less well appreciated, although there is emerging evidence that protein motions are coupled to the reaction chemistry in AdoCbl-dependent enzymes [13–17].

For ornithine 4,5-aminomutase (OAM) and lysine 5,6-aminomutase, which are both class III AdoCbl-dependent enzymes [18], a role for large-scale domain re-orientation linked to substrate binding and C–Co bond homolysis has been proposed [16,19–24]. OAM participates in the oxidative fermentation pathway converting d-ornithine to 2,4-diaminopentanoate. This intramolecular transferase reaction, involving a 1,2 shift of the amino group, is energetically and chemically challenging as it requires breakage of chemically inert C–H and C–N bonds [23,25,26]. Large-scale domain dynamics triggered by substrate binding to the pyridoxal 5-phosphate (PLP) cofactor allow OAM to overcome the energetic barrier to the reaction [19–24]. C–Co bond cleavage generates the 5′-deoxyadenosyl radical (Ado•) and cob(II)alamin radical pair. In principle, the Ado• generated may then either function as a powerful single electron oxidant or as a reversible hydrogen-abstracting catalyst [25], allowing the explicit free radical reactivity of OAM to catalyse this otherwise difficult reaction [2,19].

**Fig. 1:**
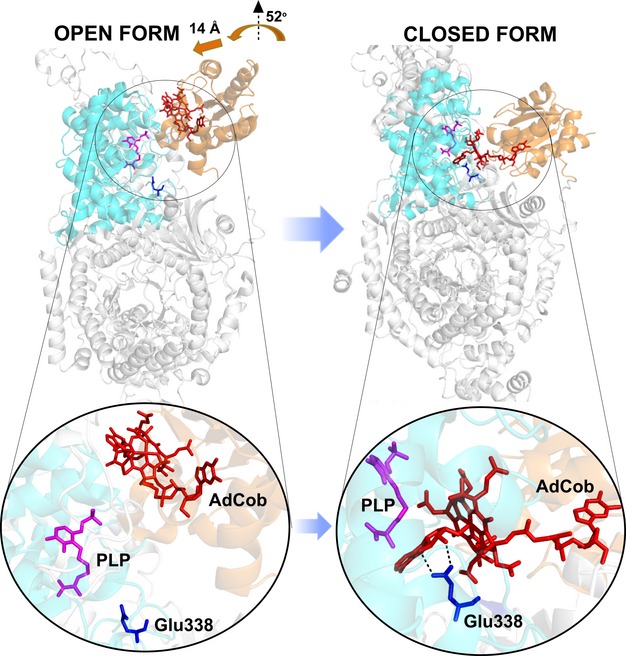
Proposed conformations of OAM based on crystal structures and molecular simulations. Open resting state and closed active state models for OAM. PLP bound to the substrate d-ornithine and adenosyl cobalamin cofactors are shown as magenta and red sticks, respectively. The Rossmann domain and TIM barrel fold are shown in brown and cyan ribbon representation. The open model is based on the published OAM crystal structure (PDB ID 3KP1), and the closed model is based on the model of Pang *et al*. [20].

Quantum mechanics/Molecular mechanics (QM/MM) calculations and molecular dynamics modelling of a ‘closed’ structure for OAM [20,21] by reference to the structure of the related enzyme glutamate mutase (GM, PDB ID 1I9C) [15,27] has suggested a mechanistic route for C–Co bond activation and subsequent homolytic rupture (Fig. 1) [19,20,23]. Later stages of the catalytic cycle of the OAM reaction have also been investigated using QM/MM methodology [22]. C–Co bond activation in the modelled ‘closed’ conformation arises through a synergy of steric and electrostatic effects arising from closer interaction with a proposed 5-deoxyadenosyl (Ado) binding motif [20,21]. In the resting form of OAM, an internal aldimine linkage between PLP and Lys629 locks OAM in an ‘open’ or catalytically ‘poised’ conformation [20,21]. d-ornithine forms an external aldimine with PLP, which releases the internal aldimine linkage, initiating large-scale movement of the Rossmann domain that harbours AdoCbl to form a ‘closed’, catalytically ‘active’ conformation. In the ‘open’ form, Ado of AdoCbl is exposed to solvent and is 23 Å away from PLP and the eight-fold β/α Triose-Phosphate Isomerase (TPI or TIM) barrel to which it is bound [20,21]. Simulations have suggested that the AdoCbl-binding Rossmann domain approaches the active site as a rigid body, undergoing a combination of 52° rotation and a 14 Å translation to bring AdoCbl to the active site [20,21]. This reduces the distance between the PLP-bound substrate and AdoCbl to approximately 6 Å (Fig. 1), placing Ado close to the external aldimine. C–Co bond homolysis coupled with transient Ado• formation and radical transfer lead to a product-like PLP-bound radical intermediate, which undergoes rearrangement to facilitate abstraction of hydrogen from Ado in a final ‘ring opening’ step [20,22]. In the absence of further turnover, the Ado• formed then recombines with cob(II)alamin to regenerate AdoCbl in the resting form of OAM.

Evidence for these large-scale domain movements during catalysis has emerged from pulsed electron–electron double resonance (PELDOR) spectroscopy of OAM in the presence of inhibitors and substrate analogues [23,28]. PELDOR and related electron paramagnetic resonance (EPR) studies identified multiple conformational states of the OAM cobalamin binding-domain, and demonstrated that, on binding the substrate, the Rossmann domain samples the available conformational space [28]. More localized motions in addition to (or in some cases instead of) large-scale motion to activate the C–Co bond have been proposed for other AdoCbl enzymes, including methylmalonyl CoA mutase (MCM) and diol dehydratase [29–32]. In the resting state of GM and MCM, active-site reorganization enables new interactions to form between the polar Ado and negatively charged active-site residues, especially with Glu370 in bacterial MCM and Glu330 in GM. This stabilizes the AdoCbl radical pair in the dissociated state. In ethanolamine ammonia lyase, a key role for a conserved Glu287 residue in forming interactions with the 2′-OH of the ribose to facilitate radical pair formation by pseudo-rotation of the Ado group has also been reported [33–35]. In the final stage of the large-scale domain movement in OAM, Ado rotates into a so-called ‘northern’ conformation imposed by the steric constraints in the active site, forcing interaction with Glu338 of the TIM barrel domain [20,21,36]. In contrast with the ‘closed’ conformation, in which Glu338 is within hydrogen-bonding distance of the Ado ribose hydroxyl group, in the ‘open’ conformation, Glu338 is > 20 Å from the ribose moiety. Modelling suggests that this steric constraint in the closed form leads to elongation of the C–Co bond by 0.03 Å, and increased puckering of the ribose moiety. The ‘strain’ imparted on Ado and the corrin ring then facilitates homolysis of the C–Co bond [20,21].

We explored here the coupling between C–Co bond homolysis and the positioning of Glu338 close to the cobalamin cofactor as a result of large-scale domain motion in OAM. Specifically, we used targeted mutagenesis to create the OAM variants Glu338Gln (E338Q), Glu338Asp (E338D) and Glu338Ala (E338A) for investigation of the proposed role of Glu338 in linking domain motion to C–Co bond homolysis. Although previous studies on OAM showed that any substitution at the Glu338 residue decreases the C–Co bond homolysis rate due to weakening of electrostatic interactions, the exact role and involvement of Glu338 in the large-scale dynamics and thus in conformational sampling leading to C–Co bond homolysis were unexplored [36]. Here, we studied this using a combination of steady-state and stopped-flow kinetic methods, EPR spectroscopy and continuous-wave (CW) photolysis of C–Co bond homolysis as a function of solution viscosity dependence. We show that Glu338 is an electrostatic facilitator of C–Co bond homolysis in a proposed dynamic electrostatic model that links substrate binding, conformational sampling and C–Co bond homolysis. Our work places Glu338 at the core of the dynamic/electrostatic model proposed based on structural analysis of the open conformation of OAM and subsequent computational simulations of active-site closure. It also provides a mechanistic structural framework for understanding the approximately 10^12^-fold enhanced rate of AdoCbl C–Co bond homolysis in enzyme systems.

## Results and Discussion

### Enzyme turnover and inhibitor binding

**Fig. 2:**
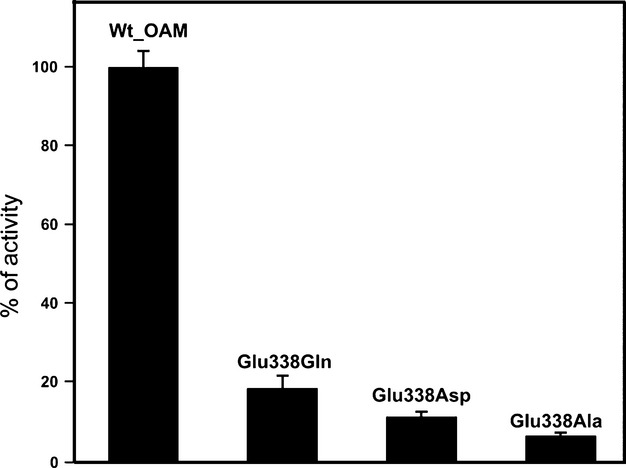
Catalytic properties of wild-type and Glu338 variant enzymes. Relative activity of the OAM variant enzymes is plotted as the percentage of catalytic turnover number (*k*_cat_) of wild-type OAM. The reaction mixtures contained 100 nm holo-OAM, 100 nm DAPDH, 0.5 mm NAD^+^ and variable concentrations of d-ornithine (0–2250 μm). A coupled spectrophotometric assay using DAPDH was used to determine the kinetic parameters for wild-type OAM and variant enzymes.

A coupled anaerobic spectrophotometric assay was used to monitor the catalytic turnover and steady-state kinetic parameters of wild-type OAM and Glu338 variants. This employed 2,4-diaminopentanoate dehydrogenase (DAPDH) as the coupling enzyme; this enzyme catalyses NAD^+^-dependent oxidative deamination of the OAM reaction product 2,4-diaminopentanoate to form 2-amino-4-ketopentonate [28,36,37]. Steady-state turnover numbers for the Glu338 variants showed a significant reduction in the turnover number, *k*_cat_, compared to wild-type OAM: as a percentage of wild-type, these reductions were 18.8% (E338Q), 11.4% (E338D) and 8.1% (E338A) (Fig. 2). The Michaelis–Menten constant, *K*_m_, for d-ornithine was similar for wild-type and the variant forms of OAM (Table 1), suggesting that replacement of Glu338 by alternative residues has little effect on d-ornithine binding; the loss of catalytic efficiency (*k*_cat_/*K*_m_) is therefore attributed mainly to changes in *k*_cat_. Anaerobic inhibition assays using d,l-2,4-diaminobutyric acid (DAB) as a competitive inhibitor were performed to determine the DAB inhibition constant, *K*_i(DAB)_, for each variant and wild-type OAM. The data were fitted using an equation describing competitive inhibition to find the inhibition constant. The largest variation in *K*_i(DAB)_ value relative to wild-type OAM was for the least active variant E338A (18.8 ± 2.2 μm), which is approximately 3.6 times higher than the *K*_i(DAB)_ for wild-type OAM under similar conditions (Table 1). The data indicate that *K*_i(DAB)_ (a guide to DAB binding) is not substantially affected by residue substitution. The *K*_i(DAB)_ values were used to guide subsequent stopped-flow and EPR studies using the inhibitor.

**Table 1:** Steady-state kinetic parameters for wild-type OAM and Glu338 variant enzymes. A coupled spectrophotometric assay using DAPDH and following the absorbance increase at 340 nm, corresponding to reduction of NAD^+^ to NADH, was used to determine the kinetic parameters. Kinetic assays were performed in a 1.0 mL volume at 25 °C using a 1 cm path-length cuvette. The reaction mixtures contained 100 nm holo-OAM, 100 nm DAPDH, 0.5 mm NAD^+^ and variable concentrations of d-ornithine (0–2250 μm). The anaerobic DAB inhibition assay was performed by addition of variable concentrations of DAB (0.15–250 μm) to determine the inhibition constant for DAB (*K*_i(DAB)_)

	*k*_cat_ (s^−1^)	*K*_m_ (μm)	*k*_cat_/*K*_m_ × 10^−3^ m^−1^·s^−1^	*K*_i(DAB)_ (μm)
Wild-type OAM	2.97 ± 0.01	189.3 ± 13.4	15.7 ± 1.1	5.2 ± 0.1
E338Q	0.56 ± 0.03	148.3 ± 11.6	3.8 ± 0.4	11.2 ± 0.1
E338D	0.34 ± 0.04	135.2 ± 8.2	2.5 ± 0.3	15.2 ± 1.5
E338A	0.24 ± 0.03	176.3 ± 8.1	1.4 ± 0.2	18.8 ± 2.2

The steady-state turnover studies showed that substitution of the Glu338 residue results in a large decrease in the turnover number of OAM. It has been shown previously that exchange of Glu338 for alanine in OAM reduces catalytic efficiency by 220-fold [36]. In MCM and GM, a similar exchange of an active-site glutamate residue was found to reduce catalytic turnover by 12- and 5000-fold, respectively [36,38]. Our findings are qualitatively similar to previous studies in OAM and other AdoCbl-dependent enzymes in which electrostatic residues close to the cobalamin 2′ ribose hydroxyl were targeted [36,38,39]. However, for reasons that are not clear, the extent of the loss in catalytic efficiency in the OAM variants reported here is less than that reported by Makins *et al*. [36]. In the OAM closed model, substitution of Glu338 mainly disrupts hydrogen bonding with the ribose hydroxyl groups of cobalamin. The Gln substitution has less impact compared to other variants, while the complete removal of a polar/charged side chain by the Ala substitution has a major effect on catalytic turnover. As the variants were designed not to perturb the interactions in the more open conformations, we infer that the observed effects on *k*_cat_ are a result of altered geometries and/or electrostatics in the closed conformation of OAM.

### Reaction chemistry in the OAM Glu338 variants inferred from UV-visible spectroscopy

The catalytic activity of wild-type OAM and the Glu338 variants were measured by monitoring anaerobically (<1 p.p.m. O_2_ content) the UV-visible spectral changes of holoenzyme when titrated against the substrate d-ornithine or the inhibitor DAB [23,28,37]. The UV-visible spectrum of holo-OAM shows overlapping contributions to the absorbance spectrum from both AdoCbl and PLP cofactors. Despite this complexity, inhibitor or substrate binding to holo-OAM under anaerobic conditions induces well-characterized spectral changes that may be attributed to chemical changes in the enzyme active site, as reported for wild-type OAM. Binding of d-ornithine to holo-OAM initiates trans-imination, as indicated by a decrease in the absorbance shoulder at 416 nm, which corresponds to PLP in the internal aldimine state; a coupled absorbance peak also appears at 425 nm [23,28,37]. The UV-visible spectrum also shows a decrease in absorbance at 528 nm (homolysis of the AdoCbl C–Co bond), accompanied by a smaller increase in absorbance at 470 nm (formation of cob(II)alamin). A larger fraction of holoenzyme undergoes AdoCbl homolysis upon binding of the inhibitor DAB, compared to d-ornithine, causing a more pronounced decrease in the absorbance at 528 nm on reaction with DAB.

**Fig. 3:**
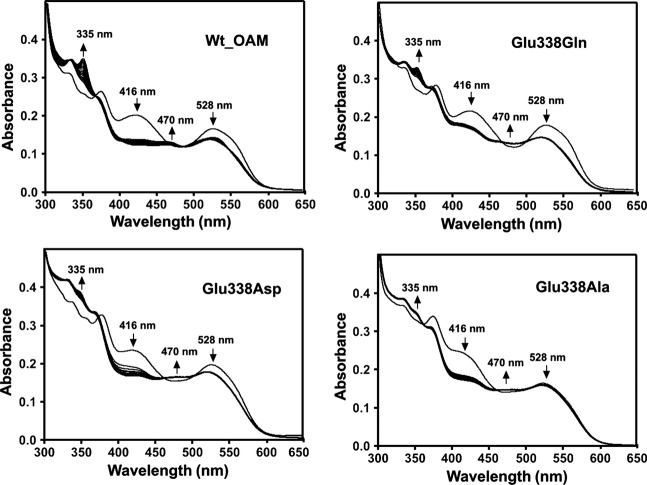
Change in UV-visible spectra of holo-OAM and Glu338 variant enzymes induced by binding of the substrate d-ornithine under anaerobic conditions. The holoenzyme solution contained 15 μm OAM, 15 μm PLP and 15 μm AdoCbl in 100 mm NH_4_-EPPS buffer, pH 8.5 (total volume 1 mL). Spectral changes for holo-OAM were recorded at 25 °C at 0 and 10 s, and then at every 60 s up to 25 min following addition of 2.5 mm d-ornithine. The arrows indicate the direction of absorbance change over time. The absorbance decrease at 528 nm reflects homolysis of the AdoCbl C–Co bond, the absorbance increase at 470 nm reflects cob(II)alamin formation, and the decrease in the absorbance shoulder at 416 nm corresponds to trans-imination induced by d-ornithine binding to PLP.

**Fig. 4:**
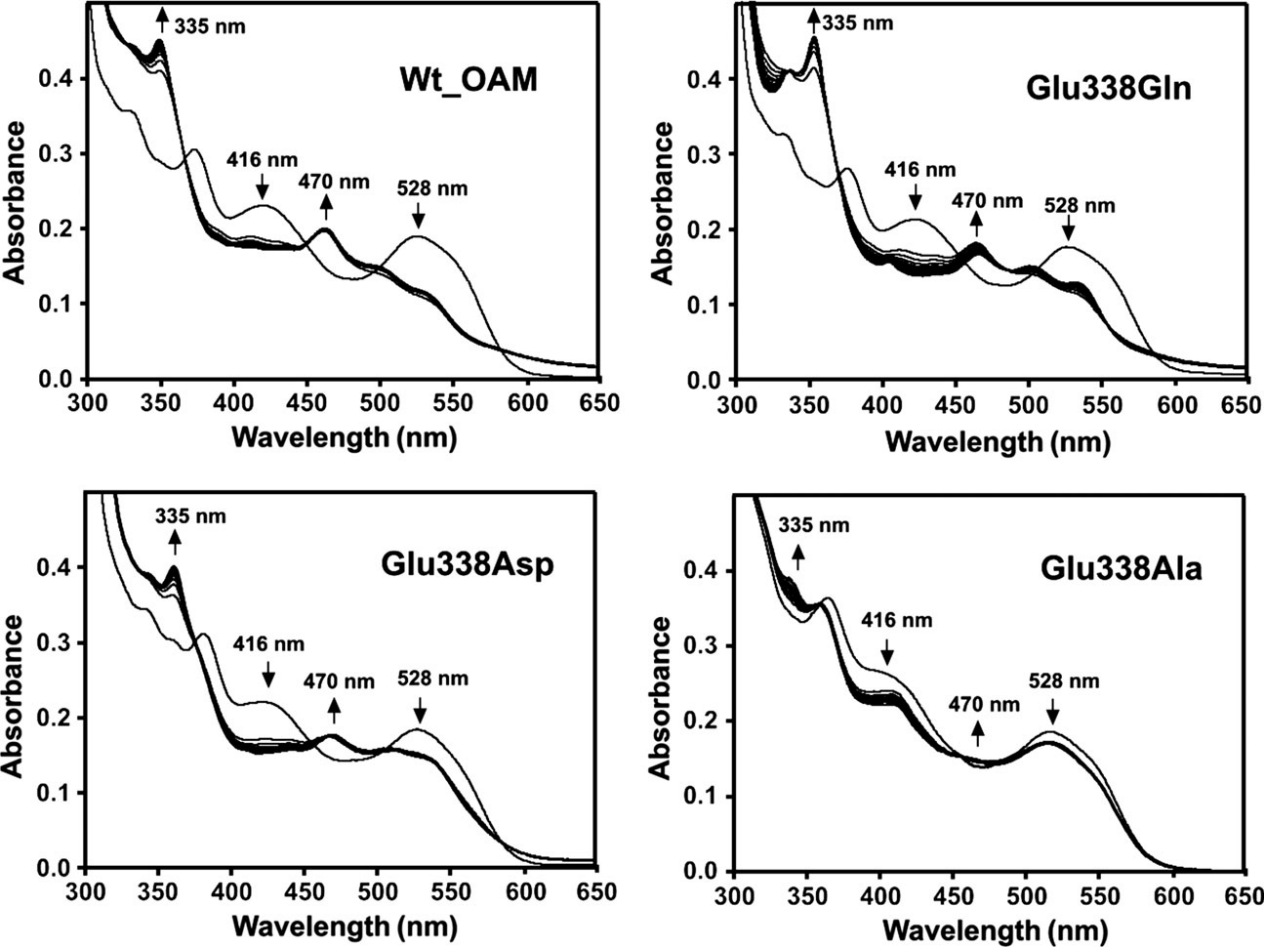
Change in UV-visible spectra of holo-OAM and Glu338 variant enzymes induced by binding of the inhibitor d,l-2,4-diaminobutryic acid (DAB) under anaerobic conditions. The holoenzyme solution contained 15 μm OAM, 15 μm PLP and 15 μm AdoCbl in 100 mm NH_4_-EPPS buffer, pH 8.5 (total volume 1 mL). Spectral changes for holo-OAM were recorded at 25 °C at 0 and 10 s, and then at every 60 s up to 25 min following addition of 2.5 mm DAB. The arrows indicate the direction of absorbance change over time. The absorbance decrease at 528 nm reflects homolysis of the AdoCbl C–Co bond, the absorbance increase at 470 nm reflects cob(II)alamin formation, and the decrease in the absorbance shoulder at 416 nm corresponds to trans-imination induced by DAB binding to PLP.

Similar spectral changes to those for wild-type OAM were also observed for the E338Q, E338D and E338A variants, but, in contrast with wild-type OAM, absorbance changes at 470 and 528 nm were found be less pronounced for the variant enzymes (Fig. 3) [23,28,37]. Absorbance changes at 416 nm, reflecting external aldimine formation, were not affected in the variant enzymes. The ability to form cob(II)alamin as a result of AdoCbl C–Co bond homolysis may be quantified from the spectral changes at 528 nm. The extent of cob(II)alamin formation inferred from these spectral changes was found to decrease in the order wild-type OAM > E338Q > E338D > E338A. This trend correlates with the variation in observed *k*_cat_ values across the OAM enzymes (Table 1). These differences are probably not associated with binding affinity, as the reactions were performed using substrate concentrations more than ten times the observed *K*_m_ values measured in steady-state assays. As trans-imination and external aldimine formation were only affected to a minor extent, we attribute the changes in *k*_cat_ following directed mutagenesis of residue Glu338 to a compromised ability to cleave the AdoCbl C–Co bond. In support of this observation, absorbance changes at 528 nm were only visible in the presence of DAB for the least active (E338A) variant (Fig. 4). Putting aside the quantitative differences in relative peak heights, the absence of observable differences in the peak positions (i.e. wavelength) across the series of variant enzymes confirms that the catalytic mechanism is unaltered in the variant enzymes compared with wild-type OAM. This is an important finding, as mutagenesis in principle may allow radical escape/quenching. We therefore conclude that the chemical nature of the reaction coordinate for the wild-type and variant forms of OAM is essentially unaltered.

### Radical intermediates observed by EPR spectroscopy

**Fig. 5:**
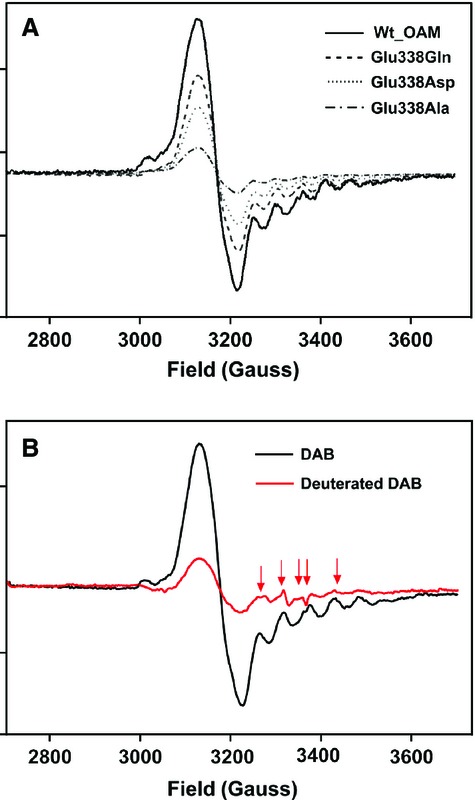
Continuous-wave EPR spectra of wild-type OAM and Glu338 variant enzymes. (A) EPR spectra showing the relative amount of paramagnetic species formed for wild-type OAM and variant enzymes in the presence of inhibitor DAB. (B) Perturbations in the CW EPR spectra of the E338Q variant when mixed with deuterated DAB ([2,4,4-^2^H_3_]-d-2,4-diaminobutyric acid) (spectrum shown in red, with arrows to indicate the corresponding spectral changes) instead of DAB (spectrum in black). The holoenzyme solution contained 250 μm OAM, 250 μm PLP and 250 μm AdoCbl in 100 mm NH_4_-EPPS buffer, pH 8.5. DAB (10 mm) was added to the holoenzyme, and samples were loaded into EPR tubes and frozen in liquid nitrogen after 5 min incubation.

EPR spectroscopy may be used to quantify, in relative terms, the extent of C–Co bond homolysis across the enzyme series following incubation with DAB inhibitor [23,28]. EPR spectroscopic characterization of wild-type OAM and the Glu338 variants in the presence of DAB therefore enabled relative quantification of AdoCbl C–Co bond homolysis and estimation of the amount of low-spin Co(II) and product-like organic radical intermediates formed. Under anaerobic reaction conditions, holo wild-type OAM or Glu338 variants were mixed with DAB, and after 5 min incubation, samples were frozen in liquid nitrogen and EPR spectra were recorded at 20 K [23,28]. The characteristic EPR spectrum of wild-type OAM in the presence of DAB provided clear evidence for spin coupling between cob(II)alamin species and organic radicals based on hyperfine splitting of the *g*_*||*_ line in the high-field region (Fig. 5A). The EPR spectrum of wild-type OAM was visually similar to that for the strong Co(II) and product radical-coupled spin system that exists in AdoCbl-dependent class I mutases [23,40,41]. A requirement for this strong coupling is the close positioning between Co(II) and the product radical (< 6 Å), which is possible only through formation of the closed conformational state. Although Glu338 variants showed similar paramagnetic spectra, the extent of radical formation is lower (Fig. 5). As for wild-type OAM, hyperfine splitting was observed for the Glu338 variants. The most active variant (E338Q) showed clear perturbation of the hyperfine splitting upon reaction with the triple deuterated inhibitor [2,4,4-^2^H_3_]-d-2,4-diaminobutyric acid (Fig. 5B). This indicates that the organic radical is derived from the inhibitor. Isotopic perturbation was not observed for the other variants (which have relatively weaker EPR spectra), as there was a large signal decrease associated with this type of kinetic isotopic effect measurement. In summary, the EPR data indicate that C–Co bond homolysis is compromised to varying extents in the variant enzymes relative to wild-type OAM.

### CW photolysis of the cobalamin C–Co bond in the ‘open’ conformation

**Fig. 6:**
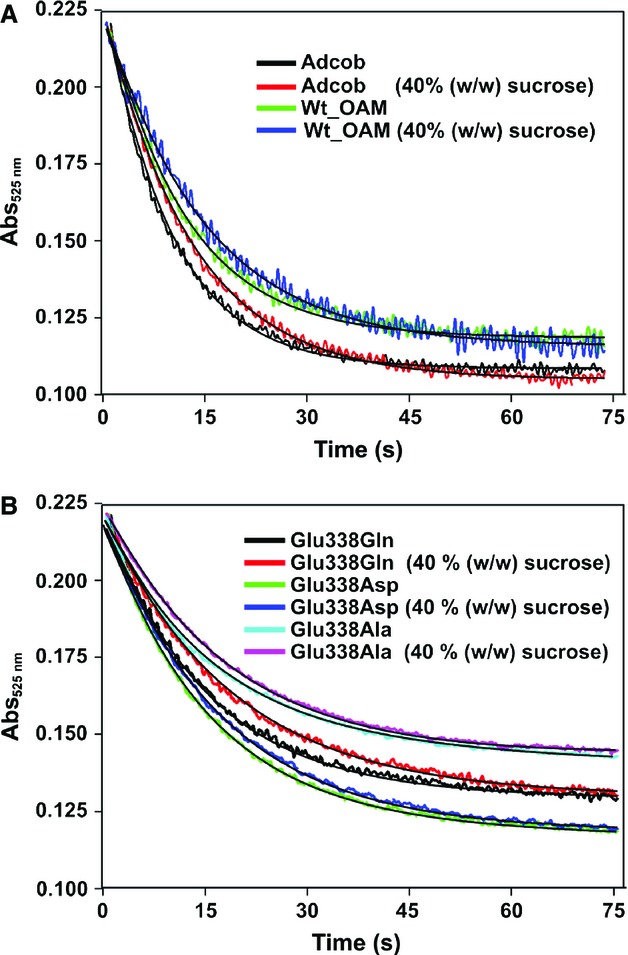
The solvent viscosity dependence on C–Co bond photolysis. Anaerobic CW photolysis experiments were performed using a stopped-flow instrument, exposing free or enzyme-bound AdoCbl to the entire emission spectrum of a 150 W Xe arc lamp (light intensity at the sample cell was 300 ± 10 μmol·s^−1^·m^−2^). Absorbance changes at 525 nm were followed using a photodiode array detector. (A) The CW photolysis transient at 525 nm for wild-type OAM when the sucrose concentration was increased to 40% w/w. (B) The corresponding transients in the E338D and E338Q variant enzymes. The photolysis rates were plotted against viscosity, and the values are fitted with an exponential decay function to determine the homolysis rate decay constant. The individual photolytic rates and homolysis rate decay constants for wild-type OAM and Glu338 variants are shown in Table 2.

Anaerobic CW photolysis experiments were performed in a stopped-flow instrument to investigate the kinetics C–Co bond homolysis in the ‘open’ conformations of OAM and the Glu338 variants. Samples were exposed to the entire emission spectrum of a 150 W Xe arc lamp, and absorbance changes at 525 nm were followed using a photodiode array detector. Absorbance data (525 nm) were described by a single-exponential equation, from which the relative rates of C–Co bond photolysis in free and enzyme-bound AdoCbl were obtained (Fig. 6). C–Co bond photolysis is similar when AdoCbl is bound to OAM (9.01 ± 0.04 × 10^−2^ s^−1^) compared with free AdoCbl (9.42 ± 0.08 × 10^−2^ s^−1^) (Table 2). The CW photolysis rates for the Glu338 variants were essentially identical to that for wild-type OAM.

**Table 2:** CW photolysis of free and enzyme-bound AdoCbl and viscosity dependence on C–Co bond homolysis. Anaerobic CW photolysis experiments were performed using a stopped-flow instrument, exposing free or enzyme-bound AdoCbl to the entire emission spectrum of a 150 W Xe arc lamp (light intensity at the sample cell was 300 ± 10 μmol·s^−1^·m^−2^). Free or enzyme-bound AdoCbl (25 μm) was taken in both syringes and rapidly mixed into the reaction chamber, and absorbance changes at 525 nm were followed using a photodiode array detector. The photolysis rates were plotted against viscosity, and the data were fitted with an exponential decay function to determine the homolysis rate decay constant

	Rate of CW bond homolysis × 10^−2^ s^−1^ (0% w/w sucrose)	Rate of CW bond homolysis × 10^−2^ s^−1^ 40% w/w sucrose)	Homolysis rate decay constant (cP^−1^)
AdoCbl	9.42 ± 0.08	8.85 ± 0.05	0.39 ± 0.02
Wild-type OAM	9.01 ± 0.04	8.10 ± 0.11	0.57 ± 0.04
E338Q	8.97 ± 0.08	7.93 ± 0.23	0.56 ± 0.04
E338D	8.83 ± 0.11	7.83 ± 0.15	0.48 ± 0.05
E338A	8.78 ± 0.05	7.72 ± 0.15	0.50 ± 0.04

**Fig. 7:**
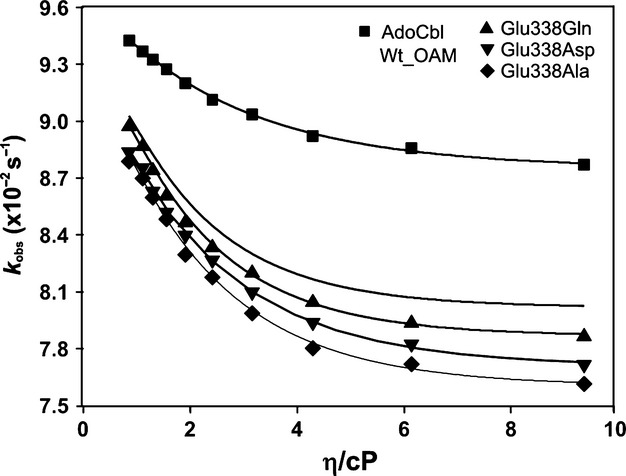
Solvent viscosity dependence on continuous-wave C–Co bond photolysis. Holo enzyme (25 μm) was taken in both syringes and rapidly mixed in an anaerobic stopped-flow instrument, exposing free or enzyme-bound AdoCbl to the entire emission spectrum of a 150 W Xe arc lamp (light intensity at the sample cell was 300 ± 10 μmol·s^−1^·m^−2^), and absorbance changes at 525 nm were followed using a photodiode array detector. The photolysis rates were plotted against viscosity, and the data were fitted with an exponential decay function to determine the homolysis rate decay constant (Table 2).

To investigate the influence of protein dynamics on the kinetics of C–Co bond homolysis, CW photolysis was performed across a range of solution viscosities (Fig. 7). Fitting the observed absorbance changes to an exponential function provided rates of C–Co bond homolysis as a decay rate constant per unit of viscosity for free and enzyme-bound AdoCbl (Fig. 7 and Table 2). The decay rate constant for wild-type OAM (0.39 ± 0.02 cP^−1^) was lower for free AdoCbl compared to OAM-bound AdoCbl (0.57 ± 0.04 cP^−1^), indicating moderate stabilization of the C–Co bond against photolysis in the enzyme. The moderate destabilization derives from the recombination events in the enzyme bound to AdoCbl, where the Ado• radical is constrained by the protein structure and cannot ‘escape’ and less cob(II)alamin appears to be formed, constraints that are completely absent in free AdoCbl in solution. The decay rate constants for the Glu338 variants were comparable with that for wild-type OAM (Table 2), suggesting that the dynamics of C–Co bond homolysis are not affected by modification of Glu338.

Previous CW photolysis measurements with ethanolamine ammonia lyase and GM led to the proposal that C–Co bond breakage was facilitated by localization of a mobile Glu residue close to the ribose moiety of the cofactor [33,42]. The modelled ‘closed’ conformation of OAM was based on the ground-state structures of GM and ethanolamine ammonia lyase, and in this conformation, one may expect C–Co bond breakage to be facilitated by the close proximity of Glu338 to the ribose moiety. Evidence for such a role is provided by EPR studies of the ‘closed’ conformation following DAB binding (see above). However, the CW photolysis measurements were performed in the absence of DAB or substrate, and OAM is therefore locked in the more ‘open’ conformation. Consequently, the potentiating effects of Glu338 localization on the rate of C–Co bond homolysis are not realised in the more ‘open’ conformation, which explains why the rate of C–Co bond homolysis is unchanged across the series of OAM wild-type/variant enzymes. The data indicate that the effects of Glu338 in potentiating C–Co bond homolysis are restricted to the ‘closed’ conformation of OAM that forms on binding ornithine or the substrate analogue DAB.

### Enzyme dynamics and C–Co bond homolysis in single-turnover stopped-flow studies

**Fig. 8:**
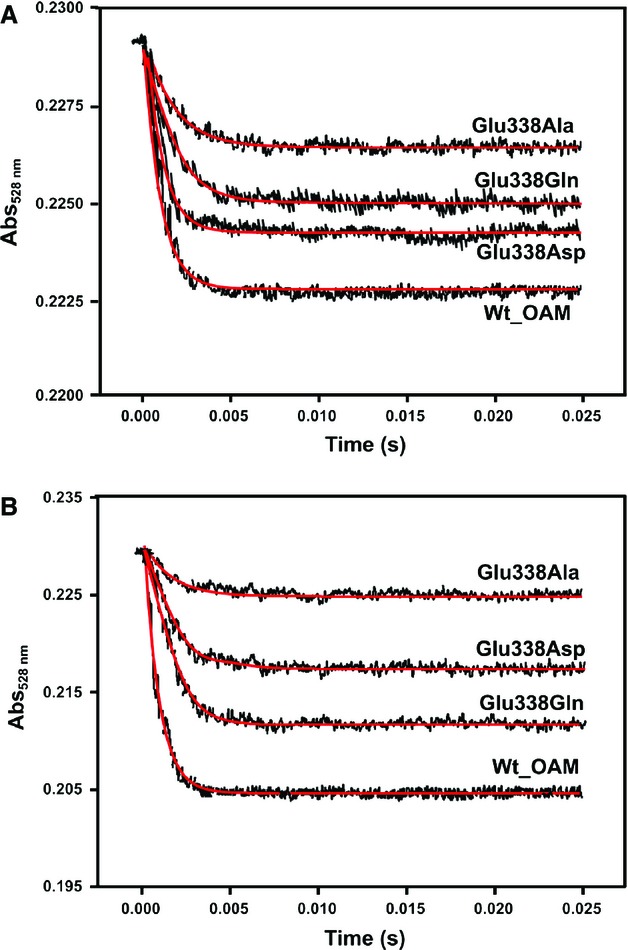
Anaerobic stopped-flow measurement of C–Co bond homolysis for wild-type OAM and OAM variants. Stopped-flow absorbance changes following mixing of holo-OAM and variant enzymes (50 μm before mixing) with d-ornithine (5 mm before mixing) (A) or DAB (5 mm before mixing) (B) under anaerobic conditions at 25 °C. The absorbance change at 528 nm reflects AdoCbl C–Co bond homolysis. Between 15 and 20 traces were averaged at each wavelength, and fitted to a single-exponential equation to obtain the homolysis rate constants (Table 3).

Rapid mixing of wild-type OAM and variant holoenzymes with either substrate (d-ornithine) or inhibitor (DAB) in a stopped-flow instrument resulted in absorbance changes at 528 nm. Observed rate constants obtained from analysis of absorbance transients at 528 nm using a single exponential function (Fig. 8) reflect Co(II) formation [23,28]. The OAM variants with the highest rate constants for C–Co bond homolysis (Table 3) were least compromised in steady-state turnover. The amplitude of the absorbance change at 528 nm was found to decrease in the order wild-type OAM > E338Q > E338D > E338A. After the external aldimine formation, this suggests poorer coupling of domain dynamics with homolysis/Co(II) formation in the Glu338 variants. We infer that conformational sampling of the mobile cobalamin-binding domain occurs in the Glu388 variants, but that, in adopting the ‘closed’ conformation, C–Co bond homolysis is compromised. The population of the ‘closed’ conformation has an associated lifetime; each time the ‘closed’ conformation is populated, there will be a higher probability of bond homolysis in wild-type OAM relative to the variant forms because of the electrostatic interactions formed between Glu338 and the cobalamin cofactor that facilitate bond homolysis. A similar electrostatic ‘trigger’ for C–Co bond homolysis is known to occur in other cobalamin-dependent enzymes [33,42], albeit not coupled to the large-scale domain motions seen in OAM.

**Table 3:** Pre-steady-state measurements and viscosity dependence of C–Co bond homolysis for wild-type OAM and variant enzymes. Stopped-flow absorbance changes at 528 nm were monitored for up to 0.25 s to follow AdoCbl C–Co bond homolysis by mixing holo-OAM or holo-OAM variants (50 μm before mixing) with d-ornithine (5 mm before mixing) or with DAB (5 mm before mixing) with increasing solvent viscosity. Between 15 and 20 traces of absorbance changes at 528 nm were averaged, and used to fit to a single-exponential equation

Enzyme	DAB (s^−1^) at 0% w/w sucrose	DAB (s^−1^) at 40% w/w sucrose	Ornithine (s^−1^) at 0% w/w sucrose
Wild-type OAM	1157 ± 24	560 ± 19	1072 ± 39
E338Q	920 ± 19	560 ± 44	694 ± 34
E338D	795 ± 14	540 ± 22	608 ± 28
E338A	621 ± 54	Not determined	564 ± 25

**Fig. 9:**
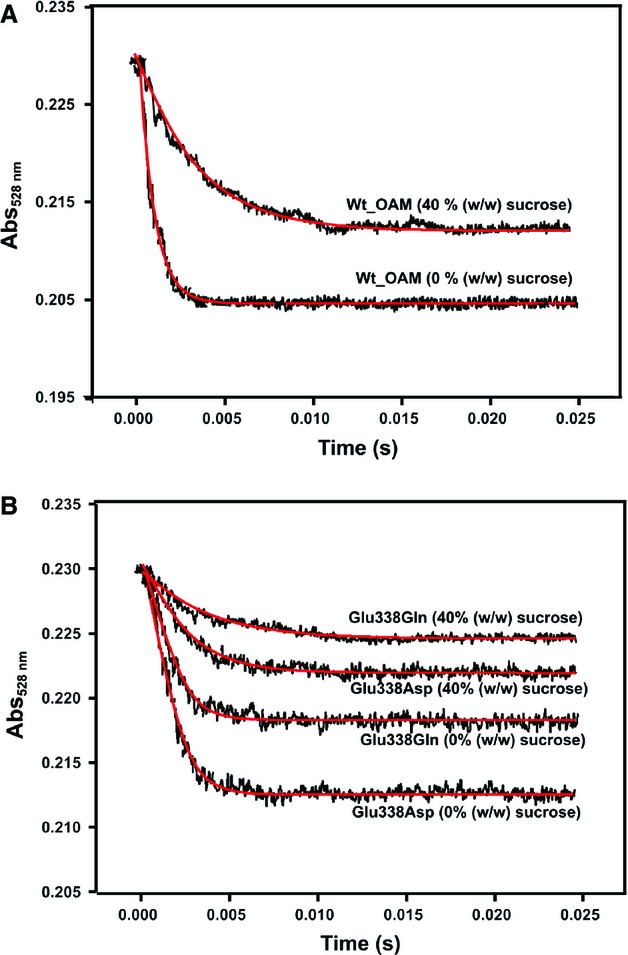
The dependence of solvent viscosity on inhibitor-initiated C–Co bond homolysis for wild-type OAM and Glu338 variants. Stopped-flow absorbance changes following mixing of holo-OAM and variant enzymes (50 μm before mixing) with DAB (5 mm before mixing) under anaerobic conditions at 25 °C. The absorbance change at 528 nm reflects AdoCbl C–Co bond homolysis. (A) Absorbance transient at 528 nm for wild-type OAM when the sucrose concentration was increased to 40% w/w. (B) Corresponding transients for the E338D and E338Q variant enzymes. Between 15 and 20 traces were averaged at each wavelength, and fitted to a single-exponential equation to extract the observed rate constants (Table 3).

Further insight into how dynamics influence the rate of C–Co bond homolysis was obtained from viscosity dependence studies of the rate of C–Co bond homolysis. Anaerobic holoenzyme was prepared in the dark in solutions containing varying quantities of sucrose. The absorbance change at 528 nm was monitored after mixing OAM with the inhibitor DAB (Fig. 9). For wild-type, the rate of C–Co bond homolysis decreased approximately twofold when the sucrose concentration was increased to 40% w/w or 6.15 cP. The decreases were approximately 1.64- and 1.47-fold for the E338Q and E338D variants, respectively (Table 3). The amplitude of the 528 nm absorbance changes was less for the variants, reflecting the extent of C–Co bond breakage. This made it impossible to perform accurate rate measurements for the E338A variant as a function of solution viscosity.

**Fig. 10:**
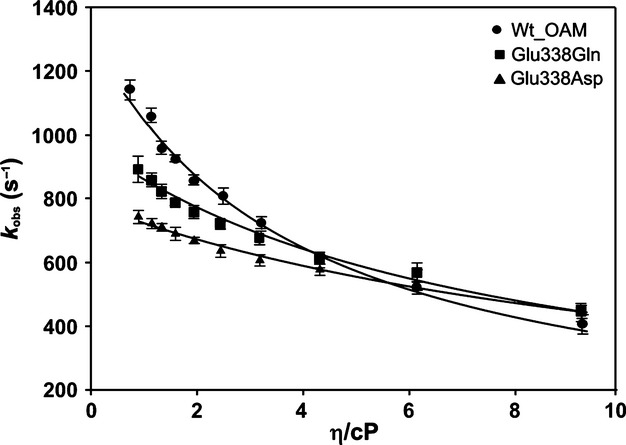
Dependence of DAB-initiated C–Co bond homolysis rate constants on solvent viscosity. The individual rates for DAB-initiated C–Co bond homolysis are plotted against varying solvent viscosity for wild-type OAM and Glu338 variants. Data were fitted to a combined Kramer–Eyring equation to calculate the contribution of protein friction to the total friction of the system (Table 2).

**Fig. 11:**
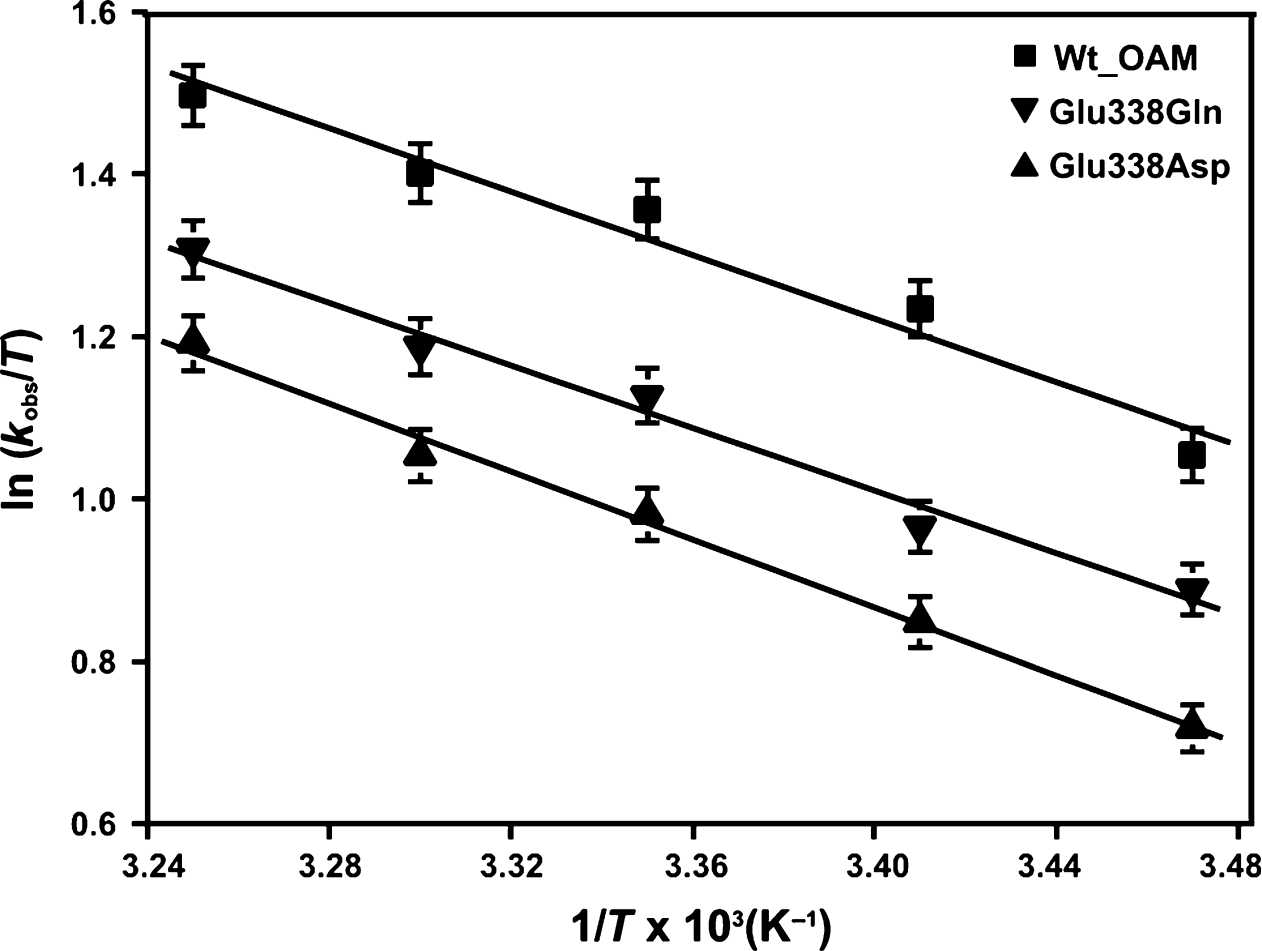
Temperature dependence and Eyring plot for DAB-induced C–Co bond homolysis in the wild-type OAM and Glu338 variants. Eyring plots of ln (*k*_obs_/*T*) versus 1/*T* for C–Co bond homolysis induced by DAB in the wild-type OAM and Glu338 variants. Data were fitted to the Eyring equation, and the activation enthalpies, Δ*H*^‡^, and activation entropies, Δ*S*^‡^, are shown in Table 4.

Kinetic data from the viscosity dependence measurements were analysed using a combined Kramer–Eyring equation [42] to understand the contribution of protein friction to the total friction of the system:


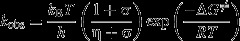


where σ, in units of viscosity, is the contribution of the protein friction, η is the absolute viscosity, *k*_B_ is the Boltzmann constant, *h* is Planck's constant, *T* is the absolute temperature, *R* is the universal gas constant, and Δ*G*^≠^ is the activation free energy for bond homolysis (Fig. 10). The activation free energies for C–Co bond homolysis for wild-type and the Glu338 variants were determined from an Eyring plot by following the rate of homolysis in the presence of inhibitor between 35 and 15 °C (Fig. 11 and Table 4). A reduction in viscosity dependence was observed for E338Q and E338D variants (σ = 7.76 ± 0.70 and 12.21 ± 0.72, respectively) compared to wild-type OAM (σ = 3.82 ± 0.63) (Table 4).

**Table 4:** Thermodynamic parameters of inhibitor (DAB)-induced C–Co bond homolysis for wild-type OAM and Glu338 variants. The enthalpies of activation, Δ*H*^‡^, and the entropies of activation, Δ*S*^‡^, were calculated by fitting the temperature dependence data to the Eyring equation. The data from the viscosity measurements were fitted using a combined Kramer–Eyring equation to calculate the contribution of protein friction (σ) to the total friction of the system

	*ΔH*^‡^ (kJ·mol^−1^)	*ΔS*^‡^ (J·mol^−1^·K^−1^)	Δ*G*^‡^ (kJ)	σ (cP)
Wild-type OAM	15.61 ± 1.81	−134.3 ± 19.1	55.7 ± 3.1	3.82 ± 0.63
E338Q	15.68 ± 1.13	−135.8 ± 19.3	56.2 ± 4.3	7.76 ± 0.70
E338D	17.03 ± 0.82	−132.4 ± 19.5	56.5 ± 1.1	12.21 ± 0.72
E338A	Not determined	Not determined	Not determined	Not determined

**Fig. 12:**
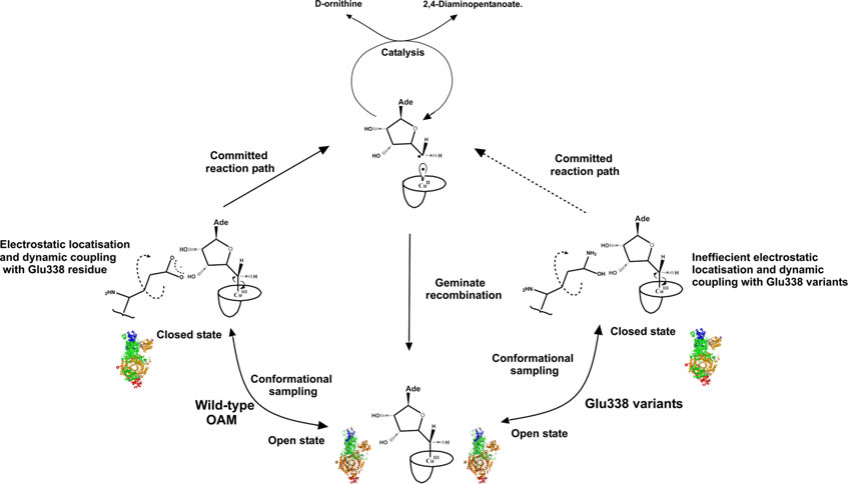
The electrostatic and AdoCbl dynamic coupling model for OAM catalysis. Upon substrate binding, OAM undergoes conformational sampling to achieve a catalytically competent closed state. In the closed state, the necessary electrostatic interactions and dynamic coupling sufficient to homolyse the C–Co bond are provided by the Glu338 residue. These interactions are compromised by the site-specific mutation for Glu338 variants.

Viscosity measurements are commonly used to identify the extent and role of protein dynamics in individual reaction steps in enzyme catalysis [17,42–44]. The viscosity measurements indicate that protein motion is coupled kinetically to the rate of C–Co bond homolysis in both wild-type and variant forms of OAM. The ‘misfiring’ or increased uncoupling of C–Co bond homolysis during conformational sampling in the variant forms of OAM may be attributed to the absence of the electrostatic trigger Glu338 that assists bond homolysis. Consequently, the extent and rate of bond homolysis are affected in the variant enzymes, ultimately compromising entry into the main catalytic cycle and affecting the observed rates of steady-state turnover (Fig. 12).

## Concluding remarks

Entry into the main catalytic cycle of OAM is dependent on conformational sampling between ‘open’ and ‘closed’ conformations triggered by the binding of substrate (or inhibitor) to PLP. Substrate/inhibitor binding breaks the internal aldimine formed between PLP and Lys629, releasing the cobalamin-binding domain and enabling it to explore both ‘closed’ and ‘open’ conformations of OAM. We have shown that C–Co bond homolysis is initiated from a closed conformation, and is accelerated through the presence of an active-site electrostatic triggering residue Glu338. Bond homolysis is coupled to conformational sampling between ‘open’ and ‘closed’ states, even when the electrostatic trigger is removed by site-directed mutagenesis. Compromised rates of bond homolysis in the variant forms of OAM are attributed to the longer time periods required to sample the closed state to facilitate C–Co bond homolysis without electrostatic assistance from residue Glu338. Our work extends understanding of the role of dynamics and electrostatics in catalysis, and shows how a combination of both (the so-called ‘dynamic/electrostatic’ model) are required to break the cofactor C–Co bond, releasing reactive adenosyl/Co(II) radicals in the first committed step for OAM catalysis. Our study provides important mechanistic insight into how AdoCbl-dependent enzymes enhance the rate of C–Co bond breakage, typically by factors of approximately 10^12^.

## Experimental procedures

### Materials

AdoCbl, PLP, d-ornithine, d,l-2,4-diaminobutyric acid (DAB) and glucose oxidase (from *Aspergillus niger*) were obtained from Sigma-Aldrich (Dorset, UK).

### Preparation of OAM variants and enzyme purification

The single mutations were introduced into the pET-OAMH2 vector harbouring OAM from *Clostridium sticklandii* using a QuikChange site-directed mutagenesis kit (Agilent Technologies, Santa Clara, CA, USA) [19,29]. The following primers (from MWG Eurofins, London, UK) were used to generate the variant forms of OAM: GLU338ALA forward primer 5′-ATTACTCCTGACGCGGGAAGAAACGTTCC-3′; GLU338ALA reverse primer 5′-GGAACGTTTCTTCCCGCGTCAGGAGTAAT-3′; GLU338GLN forward primer 5′-CAATTACTCCTGACCAGGGAAGAAACGTC-3′; GLU338GLN reverse primer 5′-GAACGTTTCTTCCCTGGTCAGGAGTAATTG-3′; GLU338ASP forward primer 5′-ATTACTCCTGACGACGGAAGAAACGTTCC-3′; GLU338ASP reverse primer 5′-GGAACGTTTCTTCCGTCGTCAGGAGTAAT-3′. The mutations were confirmed by complete plasmid DNA sequencing (MWG Eurofins, London, UK). The wild-type OAM, OAM variants and DAPDH ((2*R*,4*S*)-2,4-diaminopentanoate dehydrogenase from *Clostridium difficile*) were expressed and purified as described previously [28,37].

### Anaerobic sample preparation

Holoenzymes (wild-type OAM, OAM variants or DAPDH), reagent solution preparation and anaerobic measurements were performed in a Belle Technology anaerobic glove box (Belle Technology, Weymouth, UK) (O_2_ levels < 1 p.p.m.) under very dim light (to minimize photolysis of AdoCbl), unless otherwise stated. Buffer solutions [100 mm NH_4_-EPPS (4-(2-Hydroxyethyl)-1-piperazinepropanesulfonic acid), pH 8.5] were purged for 3 h with nitrogen, and then brought into the glove box and allowed to equilibrate for 18 h prior to use. Solid AdoCbl, PLP, d-ornithine and inhibitor (DAB) were introduced into the glove box and dissolved in anaerobic buffer. A concentrated protein sample was introduced into the glove box and gel-filtered using a 10 mL Econo-pack 10DG desalting column (Bio-Rad, Hertfordshire, UK) pre-equilibrated with anaerobic buffer. Excess oxygen was scavenged from eluted protein using glucose oxidase (13 units·mL^−1^) and glucose (10 mm) to prepare anaerobic apoenzyme samples prior to use.

### Anaerobic UV-visible spectroscopic measurements

UV-visible spectral changes of holo-OAM and variants upon binding with d-ornithine or DAB under anaerobic conditions were followed using a Cary 50 UV-visible spectrophotometer (Varian Inc., California, USA) contained in an anaerobic glove box. Wild-type OAM or variant holoenzyme solutions contained 15 μm apoenzyme, 15 μm PLP and 15 μm AdoCbl in anaerobic buffer (total volume of 1 mL). Spectral changes for holo-OAM were recorded at 25 °C at 0 and 10 s, and then at every 60 s up to 25 min following addition of 2.5 mm d-ornithine or 2.5 mm DAB.

### Steady-state and pre-steady state kinetic measurements

Steady-state kinetic parameters for wild-type OAM and its variant forms were determined using an anaerobic coupled UV-visible spectrophotometric assay with DAPDH as reported previously [28,37]. Pre-steady-state kinetic measurements were performed using an SX.17 MV stopped-flow instrument (Applied Photophysics, Surrey, UK) contained in an anaerobic glove box, essentially as described previously [28,37].

### CW EPR measurements

For CW EPR measurements, samples were prepared anaerobically as described above. Then 250 μm holo wild-type OAM or OAM variants (prepared by mixing 250 μm apoenzyme with an equimolar amount of AdoCbl and PLP in 100 mm NH_4_-EPPS, pH 8.5) were mixed with 10 mm DAB (350 μL final volume), and after 5 min incubation, samples in EPR tubes were frozen in liquid nitrogen. CW EPR spectra were measured at X-band using an ELEXSYS E500 spectrometer (Bruker, Coventry, UK) equipped with a Super High Q resonator (Bruker). Temperature control was provided by an ESR900 helium flow cryostat connected to an ITC503 temperature control unit (both Oxford Instruments, Oxfordshire, UK). The EPR spectra were recorded at 20 K as reported previously [23,28].

### CW photolysis and viscosity dependence measurements

Anaerobic CW photolysis experiments were performed in an SX.17 MV stopped-flow apparatus (Applied Photophysics) by exposing free or enzyme-bound AdoCbl to the entire emission spectrum of a 150 W Xe arc lamp (light intensity at the sample cell was 300 ± 10 μmol·s^−1^·m^−2^). Free AdoCbl (25 μm) or holo-enzyme (25 μm) was mixed rapidly into the mixing chamber, and absorbance changes at 525 nm were followed using a photodiode array detector (Applied Photophysics). Protein photo-degradation was restricted during acquisition by using a < 400 nm cut-off filter placed between the arc lamp and the sample cell. The measurements were repeated by changing the viscosity of samples by introducing varying quantities of sucrose. The photolysis rates were plotted against viscosity, and the data were fitted using an exponential decay function: *y* = A*exp(-*x*/*t*) + *y*_0_, where *A* is the amplitude of rate change, *y*_0_ is the homolysis rate in the absence of any viscogen, and *y* is the homolysis rate.

## Abbreviations

AdoCbl: adenosylcobalamin
CW: continuous wave
DAB: d,l-2,4-diaminobutyric acid
DAPDH: 2,4-diaminopentanoate dehydrogenase
EPR: electron paramagnetic resonance
GM: glutamate mutase
MCM: methylmalonyl CoA mutase
OAM: ornithine 4,5-aminomutase
PELDOR: pulsed electron–electron double resonance
PLP: pyridoxal 5′-phosphate
